# Immunity, ageing and cancer

**DOI:** 10.1186/1742-4933-5-11

**Published:** 2008-09-24

**Authors:** Evelyna Derhovanessian, Rafael Solana, Anis Larbi, Graham Pawelec

**Affiliations:** 1 University of Tübingen Center for Medical Research, Tübingen, Germany; 2 Dept. Immunology, Reina Sofia University Hospital, Córdoba, Spain

## Abstract

Compromised immunity contributes to the decreased ability of the elderly to control infectious disease and to their generally poor response to vaccination. It is controversial as to how far this phenomenon contributes to the well-known age-associated increase in the occurrence of many cancers in the elderly. However, should the immune system be important in controlling cancer, for which there is a great deal of evidence, it is logical to propose that dysfunctional immunity in the elderly would contribute to compromised immunosurveillance and increased cancer occurrence. The chronological age at which immunosenescence becomes clinically important is known to be influenced by many factors, including the pathogen load to which individuals are exposed throughout life. It is proposed here that the cancer antigen load may have a similar effect on "immune exhaustion" and that pathogen load and tumor load may act additively to accelerate immunosenescence. Understanding how and why immune responsiveness changes in humans as they age is essential for developing strategies to prevent or restore dysregulated immunity and assure healthy longevity, clearly possible only if cancer is avoided. Here, we provide an overview of the impact of age on human immune competence, emphasizing T-cell-dependent adaptive immunity, which is the most sensitive to ageing. This knowledge will pave the way for rational interventions to maintain or restore appropriate immune function not only in the elderly but also in the cancer patient.

## Introduction

Cancer is largely a disease of older people; the median age for cancer diagnosis in industrialised countries is approaching 70 years of age and is expected to increase [1]. This can be due to different reasons, such as increased duration of carcinogenesis or the susceptibility of aging cells to environmental carcinogens [2,3]. Another mechanism responsible might be reduced immune function, "immunosenescence", in the elderly. The importance of the immune system in preventing tumor formation, termed immunosurveillance, has been repeatedly shown in animal models and is supported by epidemiological evidence, such as increased frequency of certain cancer types in immunosuppressed individuals [4,5]. Here, we discuss the effects of age on immune-cell development and function, as well as the dynamics and turnover of immune cells in the elderly, and speculate as to whether many of these changes are the result of persistent challenge by a range of antigen types including cancer antigens. Immune correlates with response to vaccination and mortality and possible interventions to restore appropriate immunity and enhance responses to vaccination in the elderly are also discussed. This will be of crucial importance not only for vaccination against common pathogens such as influenza [6], but for the increasing application of immunotherapy for cancer in the elderly. Especially in the latter case, the mild or absent side effects of immunotherapy compared to chemotherapy of cancer may make this approach particularly desirable in the frail older cancer patient.

### Impact of aging on immune parameters

In elderly people, the ability to respond in a versatile way to pathogens, and potentially to cancer, is reduced due to dysregulated immunity, a state known as immunosenescence. This is characterized firstly by low numbers of naïve T cells in the peripheral blood [7] and probably also in other immune organs such as the lymph node [8], which is robustly observed in different human populations [9]. There is a corresponding reduction in the diversity of the naïve T-cell receptor (TCR) repertoire, which helps to explain the decreased ability of the elderly to resist infections to which they were not previously exposed, or to respond to the *de novo *appearance of tumor antigens. Second, although the number of memory T cells increases, there is a decrease in the diversity and functional integrity of both the CD4^+ ^and CD8^+ ^T-cell subsets [10,11] which contributes to the decreased ability of the elderly to respond adequately to re-infection or to retain memory for cancer antigens expressed by relapsing tumors. Moreover, the ability to respond appropriately to persisting infection or persistent exposure to other antigen sources, such as cancer antigens, may also be compromised, a process sometimes termed "immune exhaustion" [12].

The focus of much research on immunosenescence has been T cells, mainly because antigen presentation by dendritic cells (DCs) generally seems well-retained in the elderly [13], and because appropriate B cell [14] function depends on helper T cells. This is not to imply that non-clonotypic immunity is entirely resistant to age-related changes, or unimportant, but the alterations to natural killer (NK) cells and other innate immune cells [13] seem to be less marked than those seen with T cells. Nonetheless, it has been reported that NK cell status can predict morbidity and mortality in the elderly, emphasizing the importance of innate as well as adaptive immunity in ensuring healthy longevity and possibly cancer resistance [15]. In addition, and at least partly because of innate immune activity, the levels of pro-inflammatory factors are commonly increased in the elderly and are associated with negative effects on health [16]. Together, these changes contribute to morbidity and mortality, and are associated with poor responses to vaccination against pathogens such as those causing influenza and pneumonia [17], leading to decreased quality of life and increased health-care burdens in later life.

Studying immune cells directly *ex vivo*, as well as in culture models of ageing *in vitro*, has contributed to our understanding of age-associated changes in human T-cell-mediated immunity. However, exactly which factors are most important in immunosenescence and what causes these age-associated changes remain largely unclear. Accumulating data initially unexpectedly implicated infection with persistent herpesviruses, particularly cytomegalovirus (CMV), as contributing to the age-associated changes observed in many studies of human immune ageing [18-22]. By analogy, long-term exposure to other persistent stimulating agents, possibly parasite antigens rather than CMV, especially in developing countries, as well as dysregulated responses to autoantigens and cancer antigens, may yield similar effects. Clearly, more detailed knowledge of the reasons for immune dysfunction at advanced age and development of strategies to prevent or reverse this should contribute to maximizing healthy lifespan for the ever-growing numbers of elderly people in industrialized and, increasingly, in industrializing countries.

### Immune-cell development

**Figure 1:**
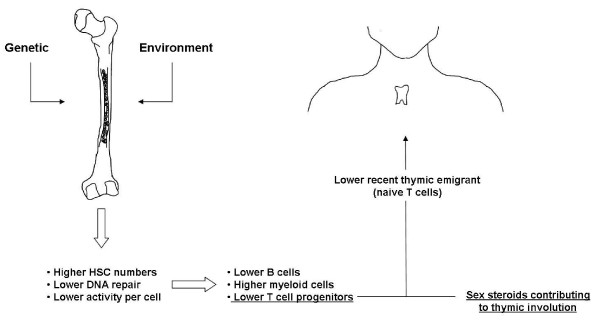
**Impact of ageing on immune cell development**. Genetic and environmental influences modulate age-associated alterations to the number and function of HSCs in the BM, resulting in skewed production of leukocytes. Reduced production of T cell progenitors, together with age-associated thymic involution, results in a relatively larger impact of age on T cells than on other leukocytes.

The cells of the immune system turn over rapidly, so compromised production or function of haematopoietic stem cells (HSCs) would affect all aspects of immunity downstream [23]. There is evidence for an association between HSC decline and longevity [24] probably due to differences in DNA damage repair [25,26]. Purified HSCs from old mice have less ability to reconstitute haematopoiesis [27]. It has been recently suggested that the observed age-associated skewing of hematopoiesis away from lymphocyte generation may be one of the factors contributing to increased cancer occurrence with age [28]. There is also a gradual decline in the ability of mouse HSCs to progress through the various B-cell differentiation stages, partly reflecting microenvironmental changes. These may involve altered production of interleukin-7 (IL-7) by stromal cells [29]. Changes at the level of T-cell progenitors also contribute to age-associated deficits in mice [30]. In any event, age is likely to impact both on HSCs and the niche environment that determines their fate (Figure 1).

Although even the very elderly can retain some degree of thymic function [31], thymic involution which represents the next hurdle that a T-cell progenitor must overcome in order to develop into a mature naïve T cell. That this could be clinically important in cancer is demonstrated in a study of age-dependent glioma-associated mortality, in which the numbers of CD8^+ ^recent thymic emigrants accounted for the effect of age on clinical outcome [32]. However, assessing immune reconstitution after HSC transplantation has demonstrated exceedingly low levels of thymic activity after the age of around 40 years [33]. Exactly why thymic involution occurs remains unclear. Because the most marked (but not the only [34] changes to thymic size and structure occur around puberty, it has been suggested that increased levels of sex hormones contribute to causing thymic involution; thymic output of naïve T cells can increase in both mice and humans following androgen ablation [35,36]. Nevertheless intrinsic defects in the commitment of haematopoietic stem cells to the lymphoid lineage [37] and the migration of T-cell progenitors into the thymus have also been implicated in this process [38]. Whatever the reason, it is clear that major age-associated changes to the thymus and greatly decreased output of naïve T cells are observed in both mice and humans (Figure 1). The elderly must therefore increasingly rely on naïve T cells already previously produced and the small numbers possibly still being produced. The presence of cancer, or particularly the treatments applied as cancer therapy, are also likely to further compromise any residual thymic function [39].

### Innate-adaptive immune interactions

**Figure 2:**
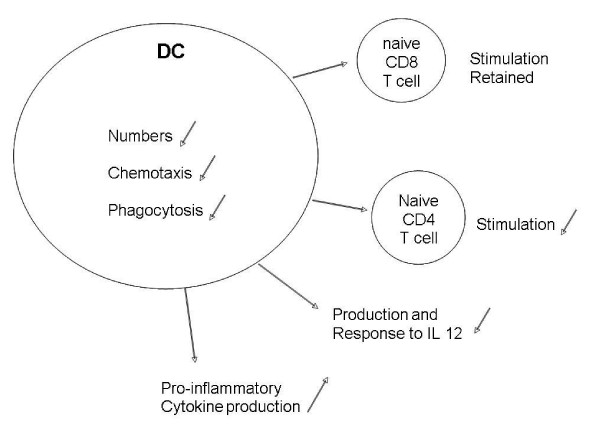
**Age-associated changes to DCs**. Reduced numbers and functions of of DCs, together with reduced numbers of naïve T cells in the periphery of aged individuals further conspire to ensure that T cell function is depressed in the elderly due to the relative decrease in T helper cell activation.

In the next stage in T-cell-dependent immunity, naïve T cells must be activated by appropriate contact with antigen-presenting cells (APCs), such as DCs (Figure 2). Although DCs seem to be only subtly different in the elderly in many respects [40], there are quantitative differences, with less peripheral blood [41] and follicular DCs [42]. Chemotaxis and phagocytosis may be impaired in DCs, as well as neutrophils [13], from the elderly [43]. DCs from young and elderly people are reported to stimulate naïve CD8^+ ^T cells equally well, but those from the elderly may fail to stimulate naïve CD4^+ ^T cells properly [43], perhaps due to altered signal transduction pathways involving phosphoinositide 3-kinase signalling [44]. In cancer, the effect of the tumor on the APC may enhance these deficits [45]. Many active immunotherapy protocols for cancer patients rely largely on DCs to be recruited to the site of vaccination and take up the vaccine antigen. Reduced DC numbers as well as their chemotactic and phagocytic activity in the elderly might represent another hurdle to be overcome in developing successful vaccination strategies for elderly cancer patients.

One very good example of the interplay between innate and adaptive immunity is illustrated by γδ-T cells [46], which also show age-associated alterations. These are able to recognize phosphoantigens present on cancer cells and have anti-tumor activities, as well as other functions, such as anti-CMV activity [47]. Their numbers rise from birth to puberty and gradually decrease again beyond 30 years of age, and they show functional alterations [48,49]. A functional analysis in young/adult and middle aged donors revealed that effector/memory γ9/δ2 CD27- T cells are increased after *in vitro *culture in the presence of specifically stimulatory isopentenylpyrophosphate and IL-2 for 10 days. In contrast, this did not occur when using cells from old subjects, confirming a lack of naive and central memory cells responding to IL-2 [50,51]. In these respects, γδ T cells behave quite similarly to conventional αβ T cells, as discussed in the next section.

### T-cell function and ageing

#### Changes at the single-cell level

To mediate an adaptive immune response, T cells must interact with functional APCs, become activated and undergo extensive clonal expansion, followed by clonal contraction. Alterations in either the number or the structure of cell-surface receptors, as well as their location in the membrane, and, once assembled, their intracellular signalling pathways, could all contribute to the final outcome of each T-cell-DC interaction. Studies have focused on alterations in signal transduction as well the formation of signalling domains (such as the immunological synapse). The interpretation of much of the published data is, however, complicated by the use of samples consisting of varying proportions of the different T-cell subpopulations in each individual, which cannot be assumed to manifest identical age-related changes.

There is no evidence for alterations to the actual structure of TCRs or co-stimulatory receptors with age, but it is likely that their assembly into functional units is compromised, leading to alterations in the assembly of the signalosome (Table 1). One major factor that influences TCR assembly is cell membrane fluidity, as first suggested many years ago [52]. However, this changes according to systemic fatty-acid availability, as illustrated by infusing lipids into young volunteers [53], and not necessarily as a result of ageing. Nonetheless, the increased levels of cholesterol which occur commonly in the elderly might well contribute to age-associated deficits in T-cell signalling [54]. High cholesterol levels are also found in some cancers, at least before the metabolic dysregulation caused by the tumor results in their lowering as the disease progresses (see, for example, [55,56]).

Although the number of TCR molecules per T cell does not seem to change with age [57], there are clear alterations in the number and balance of receptors that mediate positive or negative co-stimulatory signals. Decreased or absent expression of the most studied of these cell-surface molecules, CD28, is commonly referred to as a biomarker for immunosenescence [58], because highly differentiated CD28^- ^T cells, especially within the CD8^+ ^T-cell subset, tend to accumulate in the peripheral blood of the elderly and because CD28 expression is lost after extended culture *in vitro *as T cells undergo differentiation and enter a state of replicative senescence [58]. Studies of cultured T cells have confirmed that decreases in CD28 expression do occur within monoclonal populations as they undergo clonal expansion and progress through their finite lifespan, excluding the possibility that a small population of originally CD28^- ^cells overgrows the CD28^+ ^cells [59]. Nonetheless, the definition of such changes as biomarkers of immunosenescence remains problematic, because, for example, CD28 is downregulated by tumour-necrosis factor (TNF) [60] and upregulated by IL-12 [61], not necessarily associated with senescence. It has also been difficult to establish whether the much-investigated altered signal transduction pathways in T cells are a result of an altered balance of expression of positive and negative co-stimulatory receptors [62,63], determining the final outcome – activation, anergy or apoptosis of the T cell. Further studies on clonal populations in culture should facilitate distinguishing between changes caused by altered proportions of cell subsets and changes within the same clones at different times; a first global gene expression analysis of early and late passage T-cell clones has revealed a wide range of differentially expressed genes, including those encoding proteins involved in signal transduction and apoptosis [64]. Regarding TCR signalling in young and old T cells, even here, the differences between naïve and memory T cells, as well as between CD4^+ ^and CD8^+ ^T cells, are not well documented. For example, the well-known age-associated decrease in activation of the SRC-family protein tyrosine kinase LCK, which is one of the earliest events after TCR ligation and thought to be essential for T-cell stimulation, was recently shown to be crucial only for naïve T cells (at least in mouse CD8^+ ^T cells) [65], of which the elderly simply possess fewer.

**Table 1:** Impact of age-associated signalling changes

Features altered	Resulting altered mechanism
Cholesterol level	Signalling molecule recruitment [135]
Membrane fluidity	Protein interaction [54]
Lipid raft functions	Early signalling/immune synapse formation [135]
Calcium influx	Induction of T cell activation
Phosphatase activity	Control of negative signalling
PTK (Lck) activity	Phosphorylation of LAT [57,135]
LAT phosphorylation	Activation of LAT-associated molecules
MAP Kinase activation	Cellular activation/survival [57,135]
Cytoskeleton rearrangement	Cell-cell contact/Immune synapse formation
Translocation of transcription factors	Induction of gene expression
CD28 expression	T cell costimulation/anti-apoptotic signals [58]
KIR expression	Formation of an activation platform
KLRG-1, CD57, PD-1 expression	Induction of T cell activation [92,158]

For activation, T cells require sustained physical contact with APC. This feature is altered with age due to changes in the integrity of the membrane. Several signalling molecules fail to be activated with age, including PTK and adaptor proteins such as LAT. Calcium metabolism is altered, partly due to impaired PLCγ-1 phosphorylation. The alterations including lipid raft functions influence MAP Kinase activation and cytoskeletal rearrangements, together leading to differential activation and translocation of transcription factors such as NF-κB, NF-AT and AP-1 into the nucleus. This directly affects T cell clonal expansion due to decreased IL-2 production. Unbalanced negative signalling involving inhibitory receptors such as KIR2DL2 or Natural Killer Receptors contribute to reduce activation. PTK: protein tyrosine kinase; LAT: linker for activation of T cells; MAPK: Mitogen activated protein kinase; KIR: inhibitory killer immunoglobulin-like receptors; PLCγ-1: Phospholipase C-gamma 1.

#### Changes at the cell population level

**Figure 3:**
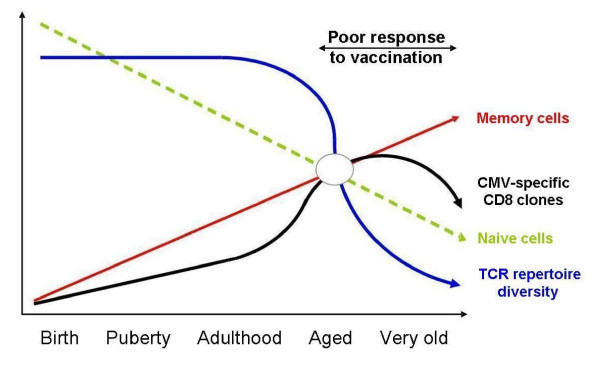
**Hypothetical model for the evolution of T cell immunity over the human lifespan**. Age-associated changes to T cell immunity result in decreasing proportions of naïve cells and increasing proportions of memory cells over the lifespan. TCR repertoire diversity is well-maintained at least until the end of the reproductive period, when repertoire shrinkage occurs rapidly. This point is reached as the number of different clonal expansions caused by persistent pathogens, as demonstrated for CMV, plateaus and then decreases. Loss of repertoire diversity in memory cells is not paralleled by their decreased numbers; on the contrary, these continue to rise as dysfunctional cells accumulate, possibly a compensatory mechanism. At this time also, responses to vaccination, as documented with influenza vaccination, are much less effective than at earlier time points.

Naïve T cells express CC-chemokine receptor 7 (CCR7) and leucocyte common antigen isoform CD45RA [66] and have long telomeres [67], whereas many memory T cells express the alternative splice variant CD45RO, have lost CCR7 and have shorter telomeres [68] suggesting an extensive proliferative history due to lack of telomere maintenance. The advent of polychromatic flow cytometry is facilitating the ever-finer *ex vivo *dissection of different T-cell subpopulations and the changes in their proportions with age. The best biomarker of immune ageing remains the relative levels of naïve T cells (defined as CD45RA^+^CD27^+^CD28^+^CD62L^+^CCR7^+^) and memory cells (with a wide range of other phenotypes [69] and which also exhibit age-related differences in TCR diversity, telomere lengths and various other factors). To mount an adequate response, a broad TCR repertoire must be maintained by ensuring the continuing presence of a diverse population of T-cell clones, but this decreases with age. At least for CD4^+ ^T cells, this may occur quite suddenly, with TCR diversity well maintained up to the age range of 60–65 years, despite the marked decrease of thymic output. However, repertoire diversity in 75–80 year olds is severely reduced [10] and probably contributes to the poor responses to infection and vaccination in this age group (Figure 3). The likely clinical implications of this are reflected in the close correlation between the diversity of the repertoire and remaining survival time in very old people [11]. One explanation for these events could be that because T cells are maintained in a state of constant turnover in the body, which is either antigen driven or homeostatic, replicative senescence (proliferative exhaustion) occurs [70]. In cell culture, CD4^+ ^T cells become more susceptible to apoptosis at this time of proliferative exhaustion [71], whereas CD8^+ ^T cells become apoptosis resistant [72]. The implication of these results is that *in vivo*, CD8^+ ^T cells would accumulate and CD4^+ ^T cells would be lost by clonal deletion, and the CD4^+^:CD8^+ ^T-cell ratio would therefore change. There is indeed evidence for the accumulation of CD8^+ ^T cells *in vivo*, and for their correlation with poor outcomes to vaccination and with mortality, as discussed in more detail below.

### Immune cell dynamics and turnover

Despite decreased thymic output of T cells, in healthy people, the overall number of T cells in the periphery remains remarkably constant at different ages, although there may be an association between slightly lower levels of both CD4^+ ^and CD8^+ ^cells and mortality at 10-year follow-up [73]. Until recently it was impossible to determine which cell subsets were proliferating and whether the rate of expansion was different in people of different ages. Pioneering work in which deuterated glucose is incorporated into dividing lymphocytes to distinguish the fraction of the pool that proliferates during the labelling time is technically challenging but very informative in humans and has now been applied to T cells, B cells and NK cells. In naïve, memory and regulatory T-cell subsets, label uptake and loss occurs rapidly but at different rates in each subset, thus indicating divergent rates of cell division, with regulatory T cells proliferating most rapidly [74]. However, the only substantial difference between young and old donors is that the CD8^+ ^memory T cells have a much longer half-life than any other subset in the elderly, but not in the young [75]. These results are consistent with an accumulation of CD8^+ ^T memory cells in the elderly. In B cells, there are also only slight differences between young and old individuals, with memory B cells turning over more rapidly than naïve B cells [76], whereas in NK cells, the production and proliferation rates were lower in the elderly than in the young [77]. Nonetheless, B cell memory may be maintained for a very long time; thus, it has been recently shown that individuals who survived the Spanish flu (H1N1) still have specific antibodies 90 years after the epidemic. These donors still possess circulating B cells that secrete antibodies that binding the respective recombinant haemagglutinin protein [78].

Given the high measured levels of peripheral T-cell turnover, it is not surprising that their proliferative potential eventually becomes exhausted; these exhausted cells are characterized by a lack of CD27, CD28 and telomerase expression [79], and by having short telomeres [80]. Memory T-cell turnover may not only result in an eventual loss of the cells, but also in an active acquisition of suppressive activity as demonstrated *in vitro *[81] and more recently *in vivo*, suggesting that development of regulatory T cells (Tregs) from terminally-differentiated CD4^+ ^cells can take place [74]. Tumor antigens as the driving force in this process might account for the commonly-observed higher levels of Tregs in cancer patients, which are believed to be a bad prognostic sign, and to interfere with immunotherapy [82]. Mechanisms therefor may include competition for antigen on the DC surface, or altered cytokine secretion patterns [59]. At least for pathogens establishing only acute infection, antigen seems not to be required for the constant turnover of specific memory T cells. In mice after clearance of acute viral infection, memory T cells persist for the lifetime of the animal and also undergo clonal expansions and accumulate even in the absence of the original antigen [83]. Thus, T-cell clonal exhaustion may be an intrinsic characteristic of adaptive immunity, which still occurs even in the absence of chronic infection [84]. However, the biological norm is likely to be chronic infection; in humans, mostly herpes viruses, especially CMV and in CMV-negative elderly individuals, Epstein-Barr virus (EBV), may be the main agents driving CD8^+ ^T cells to exhaustion [85]. In different environments, possibly where EBV and CMV are not so prevalent, other pathogens may play a similar role. Analogous phenomena of clonal exhaustion and senescence may also occur in HIV disease [86] and autoimmunity [87,88], and, as we argue here, cancer. Although the proliferative reserve of T cells is high, at least until telomere erosion reaches its critical limit (termed the Hayflick limit), being approximately 70 population doublings for CD4^+ ^T cells [70], the old idea that T-cell clonal exhaustion leads to clonal attrition and has clinical consequences has stood the test of time. The clinical consequences may be broad and impact not only on those very advanced in years, as discussed in the next section.

### Immune biomarker correlates with response to vaccination and mortality

Establishing reliable biomarkers identifying T-cell subsets and determining which are the most relevant to healthy ageing and adequate responses to vaccination requires longitudinal studies following the same individuals over time and correlating test parameters with clinical outcome (such as mortality, pathology and vaccination responses). Cross-sectional studies have the disadvantage that the young and old populations compared will have differed tremendously in their previous exposure to environmental factors of all kinds, and probably, also genetic constitution. However, so far, most human studies have been cross-sectional, and because of logistical constraints we cannot even be sure whether acute infectious complications are really more severe in the elderly due to impaired innate and adaptive immunity or due to other factors. Apparently simple questions such as the correlation of immune status with outcome of vaccination in the elderly cannot yet be answered definitively. Correlations between the presence of certain T-cell subpopulations and poor responses to vaccination against influenza virus have, however, been reported. Excessive CD8^+^CD28^- ^T cells correlate with poor responsiveness, whereas, counter-intuitively, frequencies of CD4^+ ^memory T cells, regardless of CD28 expression, do not [89]. In mouse models, the virus is cleared from the lungs more slowly in old than young mice, correlating with a delayed and decreased peak of cytotoxic T-cell production. Thus, cellular responses are crucial for controlling the virus but do not function adequately in old animals; this appears to be likely in humans too [90]. In mice, cell transfer experiments demonstrated that it was both the old environment and the old cells that contributed to the problem – that is, even young functional T cells do not respond properly when transferred to an old environment (and neither do old T cells when transferred to a young environment) [91].

**Figure 4:**
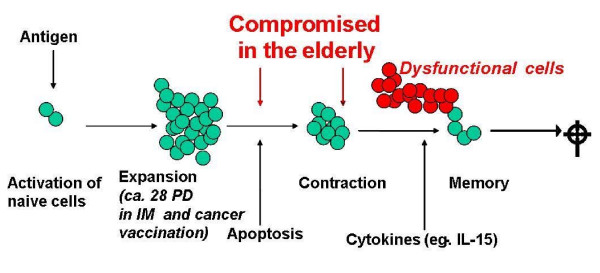
**Decreasing regenerative reserve in immunosenescence**. Hypothesis: In the elderly, dysfunctional, often CD8+ CMV- specific T cells accumulate because apoptotic pathways are compromised. Vital memory cells are eventually lost by clonal attrition (clonal exhaustion). Loss of memory cells for previously encountered pathogens contributes to morbidity and mortality. *Thus*, d*ecreased clonal heterogeneity correlates with mortality in longitudinal studies*. Because T cell homeostasis maintains constant numbers of T cells in the periphery, even the age-associated decreased thymic-output of naive cells is blocked, and the shrinkage of the naive T cell repertoire contributes to increased susceptibility to newly-encountered infectious diseases. IM, infectious mononucleosis; PD, population doublings.

The apoptosis-resistant cells that accumulate in both old mice and humans may be dysfunctional in several ways. In young mice, the number of T cells staining with soluble MHC/peptide multimers loaded with influenza-virus epitopes is very similar to the number of T cells producing interferon-γ (IFNγ) on antigen stimulation, indicating that essentially every antigen-specific cell present is able to respond to antigen. However, in old mice, the number of tetramer-positive cells exceeds the number of IFNγ-producers, indicating that a proportion of the antigen-specific cells fails to respond. This is quite similar to the situation in very elderly individuals who accumulate large clonal expansions of CMV-specific T cells, the majority of which are dysfunctional (or anergic). Longitudinal studies of elderly Swedes have established that individuals with the largest accumulations of clonally expanded CD8^+ ^T cells, most of which are CMV-specific and have a mature phenotype (CD27^-^CD28^-^CD57^+^KLRG1^+^) [92,93], have a shorter remaining survival time than people of the same age with less of these cells at baseline. In CMV-positive subjects, the number of different clonal T-cell expansions increases from middle age to old age and decreases in the terminal phase of life, resulting in a highly significant correlation between the number of T-cell clones and survival of the individual [11]. This finding suggests that eventual loss of control of CMV infection after a lifetime of immunosurveillance may indeed be a cause of mortality in this elderly population (Figure 4). Whether this will be a general finding in other populations remains to be seen. Clonal expansions of CD8^+ ^T cells are also found in old mice; whether this is antigen independent (random TCR usage; homeostatic proliferation) or antigen driven may depend on the pathogen environment [94]. Thus, although antigen driven, especially CMV-stimulated, T-cell clones may be the type most frequently found in aged humans, this finding suggests that there are multiple, independent mechanisms that can contribute to age-altered CD8^+ ^T-cell homeostasis. All these findings suggest that dysfunctional, end-stage differentiated cells may accumulate in various physiological situations [95]. To what extent this process is amplified in CMV carriers who are also cancer patients, and not only for CD8^+ ^but also CD4^+ ^T cells as well as possibly components of innate immunity [13,96] must be further investigated. This notion is consistent with the hypothesis that such CD8^+ ^cells fill the "immunological space", thereby decreasing immune competence and raising the possibility of intervening to restore appropriate immunological function [97,98]. As we learn more about the dysfunctional status of these cells, in terms of basic biochemical functions [99,100] and receptor signalling [101] we may begin to identify biomarkers that can be used in monitoring therapy success [102,103]. There is some evidence for the existence of such "exhausted" cells in cancer patients [104,105]. Whether the accumulation of dysfunctional cells can influence response to therapy, in particular to immunotherapy in cancer patients, remains to be explored; very few approaches to this question have been made so far, but a limited literature exists in animal models.

### Cancer immunity and ageing

The extent to which tumors are controlled by immunity is controversial. Nonetheless, there is clear evidence for the immunogenicity of cancer cells and large numbers of cancer immunotherapy trials have shown beyond reasonable doubt that T cells recognizing tumor antigens can be amplified in many patients. The calculated frequency of cells responding to a vaccine antigen (MAGE-3) in melanoma suggests that responding T cells must divide a large number of times to generate the observed number of responding cells and to be effective in patients [106]. This extreme requirement for clonal expansion might well be partially responsible for the lack of success of many cancer vaccination trials, due to the phenomenon of replicative senescence in T cells [70].

Limited studies in preclinical animal models have indicated age-associated alterations in tumor immunity and therapeutic responses. Thus, in a mouse breast cancer model, there are effective anti-cancer immune responses in young animals which are mostly mediated by T cells, whereas the (less effective) responses in old animals primarily rely on innate immunity [107]. This could be a general finding, because adaptive immunity is highly susceptible to deleterious changes with age, but innate responses tend to be better-preserved [108]. In fact, retention of strong inflammatory responses with age, but now in the absence of the counterbalancing and beneficial effects of the adaptive responses that they normally amplify at younger age may even enhance immunopathology and carcinogenesis. This might also help to explain the genetic component of certain cancers in which inflammation is well-established as an amplificatory factor [109]. On the other hand, tumors may be less aggressive in the elderly, as seen in breast, lung, colon and other sites in very old people and centenarians, partly because of the lower inflammatory status of "successfully aged" people, which also decreases the ability of the stromal tissue to support tumour growth and angiogenesis [110].

According to these considerations, young and old individuals will most likely require different treatment approaches, which could solve some of the problems of treating geriatric cancer patients with other, more traditional, modalities. Cancer could be less responsive to chemo- and radiotherapy in the elderly, and this might be because the immune system is less effective in the elderly, following the notion that even responses to chemo- and radiotherapy require an intact immune system [111]. In the few animal models investigated thus far, cancer immunity successful in young individuals is mostly less effective in older ones. Young mice injected with highly immunogenic tumors such as chemical- or radiation-induced sarcomas controlled the tumor better compared to old mice. In contrast, tumors with weak immunogenicity, like B16 melanoma, were more aggressive and grew better in young animals compared to old (reviewed in [112]). Sometimes, decreased efficacy in old mice can be compensated for by increasing costimulation [113,114], which is focussing efforts on improving adjuvants for the purpose of better vaccination results in the elderly in general. This may be particularly important given the possible synergistic effects of ageing and cancer on DC function, such that the relative contribution of DC dysfunction may be greater in cancer than in healthy ageing [115]. However, as with other aspects of the complex interactions between cancer and ageing, it will be difficult to generalize from one system to another. Thus, it has already been demonstrated that some immunotherapies are actually effective only in old but not young animals [116], implying that patient-individualized therapies will have to take the immunological age of the individual closely into account. For this, it will clearly be necessary to differentiate between chronological age and the "functional" age of immunity, as could be based on the constellations of immune markers making up the "immune risk profile" or the modified set of markers investigated by Hirokawa et al. mentioned above [105].

### Possible interventions and improved vaccination strategies

As discussed above, immunosenescence results from multifactorial processes that act on all components of the immune system and some approaches to reconstitute appropriate function have already been mentioned. Recent results from studies on long-lived non-human primates maintained on a calorie-reduced diet showing improved maintenance and/or production of naïve T cells, better T-cell function and reduced pro-inflammatory status [117], and the possible beneficial effects of moderate exercise [118], serve to emphasize the continued relevance of common-sense medical advice in "anti-ageing" medicine as applied to immunosenescence. Although as ageing proceeds, the T- and to some extent B-cell compartments seem to be particularly susceptible to senescence, NK cells and other components of innate immunity are also affected. As mentioned above, some limited data do point to a crucial impact of NK-cell status on resistance to infectious disease and hence healthy longevity [15], as well as being implicated in the outcome of influenza vaccination [119]. It is likely that NK cells also play a role in determining the outcome of cancer immunotherapy [103]. Clearly, it is not only the T-cell status and/or CMV status of elderly subjects that affects the outcome of vaccination [120], but also the pre- (and post-) vaccination NK-cell status [119] (although how much the latter is affected by CMV is not yet clear). It is therefore imperative that these studies are extended and repeated in different populations. Nonetheless, the most severe clinical impact is likely to remain primarily the result of loss of TCR and BCR repertoire diversity due to clonal deletions, decreased thymic and bone marrow output, and accumulations of dysfunctional cells. In addition to avoiding over-eating and ensuring that sufficient exercise is taken, what else can be done to reconstitute immunity at old age?

#### Restoring naive T-cell number and function

Given the paucity of naïve T cells in the elderly, enhancement of thymic output is likely to be a first requirement for maintaining or restoring their effective immunity. However, although in a contrived experimental situation in mice, neonatal thymi grafted under the kidney capsule exported the same numbers of T cells regardless of whether the peripheral pool was oversupplied (by thymic grafts) or undersupplied (due to neonatal thymectomy) [121], feedback mechanisms maintaining T-cell homeostasis in humans would be expected to prevent any residual thymic output due to the accumulated dysfunctional CD8^+ ^T cells present in the periphery. This may be consistent with age-associated decreases in thymic output of CD8^+ ^T cells being more marked than CD4^+ ^T cells, at least in mice [122]. Such CD8^+ ^T-cell accumulations could be reduced or eliminated by identifying ways of specifically targeting the dysfunctional subset [98]. Efforts directed at thymic reconstitution could then follow.

In mice, it was reported that transplantation of aged involuted thymi into juvenile recipients led to reconstitution of the structure and function of the thymus [123]. Moreover, transplantation of cultured thymic fragments to patients with DiGeorge syndrome who lack a functional thymus, has been carried out successfully [124] and may also be a conceivable approach to restore naive T-cell numbers in the elderly. The use of T-cell survival factors, such as IL-7, has been explored in mice and monkeys, and local delivery of this cytokine to the thymus by implantation of genetically engineered stromal cells secreting it may be a way of avoiding systemic effects [125]. Sex steroid ablation in men undergoing therapy for prostate cancer is reported to result in increased numbers of circulating naïve T cells, but this approach is obviously not generally applicable to the majority of elderly people [35]. More recently, other factors such as keratinocyte growth factor have been shown to improve thymic output in old mice [126]. Thymic epithelial cells, which are required to support thymocyte maturation, undergo apoptotic death in the aged thymus, through a pathway involving interactions between FAS and FAS ligand. Because age-associated thymic involution is reported not to occur in aged FAS-deficient mice [127], blocking this pathway locally in the thymus might also contribute to retaining thymic functionality. The same may apply to transforming growth factor-β2 (TGFβ2), as greater thymic cellularity and higher levels of naive T cells are seen in old TGFβ2-deficient mice compared to old wild-type mice [128]. T cells that have undergone clonal exhaustion after chronic viral infection also express the B7-family receptor named programmed cell death-1 (PD1), that inhibits co-stimulatory signals [129]. It has been suggested that blocking PD1-mediated signalling could lead to improved T-cell function [130].

It is too early to say whether any of these approaches can be translated to human therapy. It is possible that, among its other effects, CMV has a role in reducing thymic output: at least in clinical human HSC transplantation, output of naïve T cells is reduced by CMV infection [131]. Steps taken to reduce CMV infection might therefore also benefit naïve T-cell production.

#### Nutrition and inflammation

Interventions in nutrition, which are relatively easy to carry out, could have a larger impact on immune function than commonly appreciated, even in donors selected for very good health [132]. Vitamin E supplementation has received much attention, and was recently reported to reconstitute immunological synapse formation, especially in CD4^+ ^naïve T cells of old mice [133]. However, many nutritional interventions could be viewed more as correcting poor diet, or reducing an inappropriately rich diet, but this alone could have a major impact on immunity in the elderly. Given the notion that increased basal levels of inflammation and frailty in the elderly are intimately related, anti-inflammatory nutritional interventions could be useful [16]. Modulating lipid intake would be a possibility in this context, using, for example, conjugated linoleic acid, which can result in decreased pro-inflammatory cytokine secretion and which can increase the success rates of hepatitis B vaccination in the elderly [134]. As discussed above, the lipid environment strongly influences T-cell function, and alterations in membrane fluidity affect lipid raft and immunological synapse formation [52,135]. Human high-density lipoprotein extracts accumulated cholesterol from lipid rafts, resulting in increased TCR signal transduction and T-cell activation [136].

Use of non-dietary anti-inflammatory agents to decrease levels of IL-6, TNF and IL-1 may also assist in rebalancing immunity; to this end, it might be possible to use relatively innocuous agents, such as statins, that are already being used extensively in the elderly to treat autoimmunity and other diseases [137]. In particular, the level of IL-6 was found to be an independent predictor of mortality in longitudinal studies, which, together with a cluster of immune parameters designated the immune risk profile (IRP, see next section), constituted a super-additive risk of mortality [138]. However, the efficacy of any approach to influence inflammation is very much open to question, as inflammation also has protective effects against pathogens. From a theoretical point of view, given clear associations of IL-6, for example, with frailty [139], reducing the levels of such mediators would nonetheless be beneficial not only for improving immune status, via improved antigen presentation [140], for example, but also by its effect on many other organ systems. Given the contribution of inflammation to carcinogenesis, there may well be benefit of using anti-inflammatories in this context as well. Nutritional intervention by caloric restriction, that was shown to improve the immune response in rodents [141], has also been shown to delay T cell immunosenescence in non human primates, preserving the number and function of naive T cells [117].

#### Antigenic load

Given the initially quite unexpected impact of infection with persistent herpesviruses on immunity in the elderly [19] strategies to reduce the infectious antigenic load would seem to offer a reasonable approach to restoring appropriate immunity. The Swedish OCTO/NONA longitudinal studies [142] have revealed several changes in immune parameters predicting mortality at 2, 4 and 6 year follow-up, and summarized in what we have designated the IRP [143]. This is a cluster of immune biomarkers which may be informative in several elderly populations [144,145], as well as in other circumstances, for example, for the occurrence of infections after HSC transplantation [146] and autoimmunity [88]. An initially surprising finding was the association of the IRP in the elderly with CMV infection [147], but not with morbidity [148], which is consistent with the effect of persistent herpesvirus infections on immunity. A recent study [149] has also shown a progressive accumulation of HSV-specific T cells with a central memory phenotype in old mice. However the continuous administration of antiviral drugs did not alter the course of this accumulation of T cells. These results suggest the possibility that, at least in this experimental model, T cells expansions arise as a consequence of age-associated homeostatic disturbances rather than repeated antigenic stimulation. Nevertheless, the direct influence of CMV and other herpes viruses as a major driving force in T cell immunosenescence cannot be excluded. Thus, interventions targeting these viruses or the accumulated dysfunctional memory cells specific for them [92], may be of clinical benefit both directly against viral infection and in terms of improving responses to vaccination. The same principle may apply to other human populations in areas of differing pathogen load, for example parasitic infection, rather than herpesvirus infection [150]. In addition to pathogens, here we have argued that tumors, which are probably very common in the elderly but not clinically apparent in most, and which are immunogenic, are also likely to contribute [104]. Therefore, strategies to reduce these sources of chronic antigenic stimulation could have a general immunorestorative role on age-associated immune dysfunction.

The presence of cancer is commonly associated with compromised immune responses [95]; surgical removal of the tumour can result in restoration of immunity [151]. By analogy, reducing the antigenic load in the elderly, for example, by applying antiviral agents, might be beneficial (although some of these agents may themselves be immunosuppressive [152]. Alternatively, the failure of the immune system to entirely eliminate persistent herpesvirus infection may represent a trade-off with some actual benefits to the host; at least in mice (but only middle-aged were tested....), infection with a virus that is similar to human CMV seems to confer protection against infection with certain bacteria [153]. The mechanism for this appears to be by maintaining a higher level of pro-inflammatory mediators in the periphery, especially IFNγ; however, this could be part of the problem, protective in the young, but detrimental in the elderly. So, we may again be seeing a trade-off between pro-inflammatory status conferring protection against infection in early life but anti-inflammatory status conferring an advantage in later life [154].

#### Vaccination

One of the greatest practical health-care challenges in the elderly is to ensure that vaccinations are optimally effective. Increasing the efficacy of influenza vaccination would have an enormous impact on health and well being, but other vaccinations, including those currently under development for treating cancer [155] will also become more important in this respect. Effective vaccination may not only protect against the specific pathogen, but also may result in enhanced activity of the NK-cell system, which in turn may be associated with better specific vaccine responses [119]. A combination of improved vaccines with better adjuvants and immunostimulatory agents would be of further benefit, as the commonly applied adjuvant alum is only marginally effective in the elderly [156] and in fact mostly enhances antibody responses, whereas resistance to viruses and cancer may benefit more from enhanced cellular immunity [90].

There is great promise in using TLR ligands that strongly enhance immunization efficacy and IL-2 production [157]. The putatively dysfunctional CMV-specific CD8^+^CD28^- ^T cells that accumulate in the elderly and that seem to be anergic and apoptosis-resistant directly *ex vivo *may be restored to functional competence by culturing them with IL-2. In this respect, they behave like anergic T cells in many experimental systems, in which alternative approaches including blocking inhibitory receptors, such as PD1 mentioned above, can restore T-cell function in situations of chronic antigen exposure [158]. One option could be therefore to treat the elderly with recombinant IL-2; an early study reported that 39 elderly people given well-tolerated low-dose IL-2 just before receiving influenza virus vaccination produced higher antibody titres and were better protected than controls vaccinated without IL-2 pre-treatment [159]. A more recent study using a novel IL-2-supplemented liposomal vaccine in 48 elderly people also found better responses with IL-2 [160]. It remains extremely important to confirm and extend these studies.

The requirement for improved vaccination protocols applies not only to infectious disease but possibly also to vaccination against cancer, primarily a disease of the elderly, as illustrated by differences in responses to anticancer vaccinations in young and old mice [161]. Clearly, however, vaccination can only be effective if cells that are capable of responding are still present in the repertoire. These may be either naïve cells to be stimulated with vaccine containing novel antigens, or memory cells requiring boosting by previously encountered antigen. For naïve cells, as discussed above, the main question is whether old individuals still have any towards the end of their lives and if so, whether they are fully functional. In mice, naïve T cells from old animals do seem to be impaired [162]. CD4^+ ^T cells show decreased helper activity and IL-2 production [163], which can nevertheless be partially restored by exposure to a mixture of pro-inflammatory cytokines (IL-1, IL-6 and TNF) [164]. Thus, judicious local use of these cytokines as adjuvants might be beneficial. However, in elderly humans, there may be vanishingly few naïve cells remaining that could be targeted in this way [165].

## Concluding remarks

If the elderly must mostly rely on their memory T cells for pathogen and cancer control in later life, it becomes crucial to know whether these are retained and function normally [166], and if not, what can be done about it. In mice, depletion of the dysfunctional naïve T cells can result in their replacement by functional recent thymic emigrants [163]. This approach, coupled with thymic regeneration and HSC maintenance, could have a marked clinical impact. Better understanding of immune dysfunction in human ageing will increase the probability of discovering means to restore appropriate function and alleviate the burden of infectious disease and cancer late in life, and may possibly also benefit younger cancer patients as well. Key questions remaining are the relative importance of age-associated alterations in adaptive and innate immunity, especially NK activity, to human health and longevity; the relative roles of CMV-vs-other persistent infections, such as with parasites rather than viruses, in accelerating immunosenescence and their impact on areas of immunity other than T cell immunity; whether pathogen load and tumor antigen load act additively or, worse, synergistically, in hastening immunosenescence and, obviously, how to intervene effectively to reconstitute appropriate immune function throughout life.

## Competing interests

The authors declare that they have no competing interests.

## Authors' contributions

All authors contributed to drafting the manuscript. All authors read and approved the final manuscript.
